# Fulminant Nonocclusive Mesenteric Ischemia Just after Hip Arthroplasty

**DOI:** 10.1155/2010/250436

**Published:** 2010-03-15

**Authors:** Maria Auxiliadora-Martins, Gil Cezar Alkmin-Teixeira, Omar Feres, Olindo Assis Martins-Filho, Anibal Basile-Filho

**Affiliations:** ^1^Divisão de Terapia Intensiva, Departamento de Cirurgia e Anatomia, Faculdade de Medicina de Ribeirão Preto, Universidade de São Paulo, São Paulo, Brazil; ^2^Divisão de Proctologia, Departamento de Cirurgia e Anatomia, Faculdade de Medicina de Ribeirão Preto, Universidade de São Paulo, São Paulo, Brazil; ^3^Laboratório de Biomarcadores de Diagnóstico e Monitoração, Instituto René Rachou, Fundação Oswaldo Cruz, Belo Horizonte, MG, Brazil

## Abstract

Nonocclusive mesenteric ischemia (NOMI) is not a rare clinical entity in intensive medicine, and it can be a consequence of several clinical or surgical situations. This pathology results from reduced intestinal microvascular blood supply associated with an acute inflammatory process, culminating with bowel necrosis. This is a case on a female patient who developed immediate postsurgical NOMI following hip arthroplasty and died. Since diagnosis of this potentially fatal condition remains a dilemma, NOMI should always be considered an eventual postoperative complication in high-risk surgical patients such as elderly individuals with previous history of nicotine abuse, congestive heart failure, and essential hypertension. The present paper highlights the importance of early diagnosis and prompt adequate treatment of NOMI in subjects with diminished cardiac output and severe abdominal pain.

## 1. Introduction

The potentially fatal nonocclusive mesenteric ischemia (NOMI) is not a rare complication in intensive medicine. NOMI is defined as mesenteric ischemia with normal splanchnic vasculature. This condition can be associated with several clinical and surgical situations, and it represents approximately 20% of all the cases of acute mesenteric ischemia, with a mortality rate of about 70% [1, 2]. In intensive medicine, NOMI is a very common disruption in critically ill patients or during major surgeries, being strongly associated with a fatal outcome [1]. This pathology is generally characterized by acidosis of the gastrointestinal mucosa secondary to hypoperfusion or ischemia. The mechanisms of subjacent mucosal ischemia, which is usually caused by acute vasoconstriction resulting from splanchnic hypoperfusion of peripheral arteries, are currently being unveiled. Risk factors include low cardiac output, sepsis, use of vasoactive drugs, digitalic intoxication, hemodialysis, heart failure, abdominal surgery, or any other severe illnesses [2–4]. According to a study involving 62 cases of fatal NOMI, heart failure is considered the main risk factor [1]. Since NOMI remains a major challenge to both surgeons and intensivists [5, 6], all the abovementioned predisposing factors should be identified as risk factors for the onset of this condition, even in the case of patients of elective surgeries with normal preoperative laboratory findings [7, 8]. This work describes the case of an elderly female patient with a history of systemic arterial hypertension that developed fulminant NOMI in the immediate postoperative period of hip arthroplasty. 

## 2. Case Report

**Figure 1:**
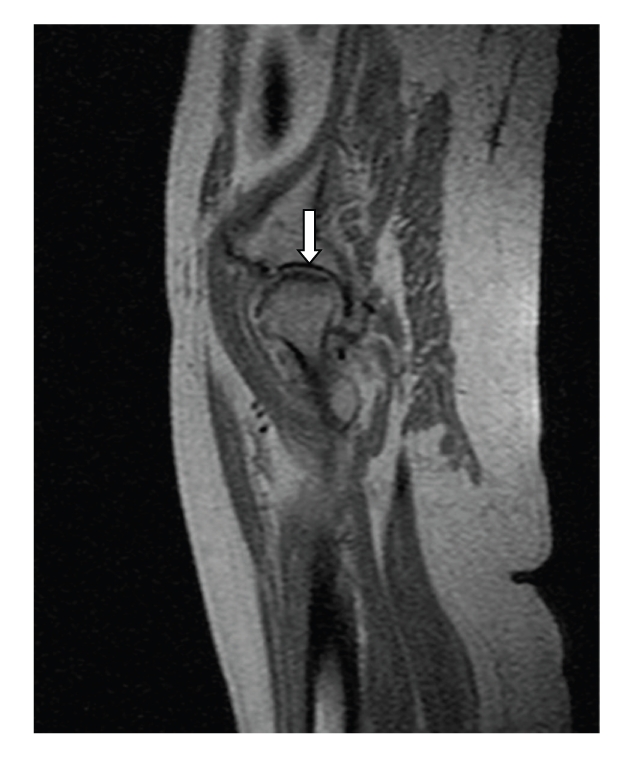
Aseptic necrosis (avascular) of the left femoral head with flattening of the subchondral bone (arrow).

This is a case report on a 77-year-old female patient who had been complaining of acute left hip pain during deambulation for 2 months. The patient had a history of essential hypertension, which was being controlled with drugs. Magnetic resonance imaging evidenced aseptic necrosis (avascular) of the left femoral head (Figure 1). 

**Figure 2:**
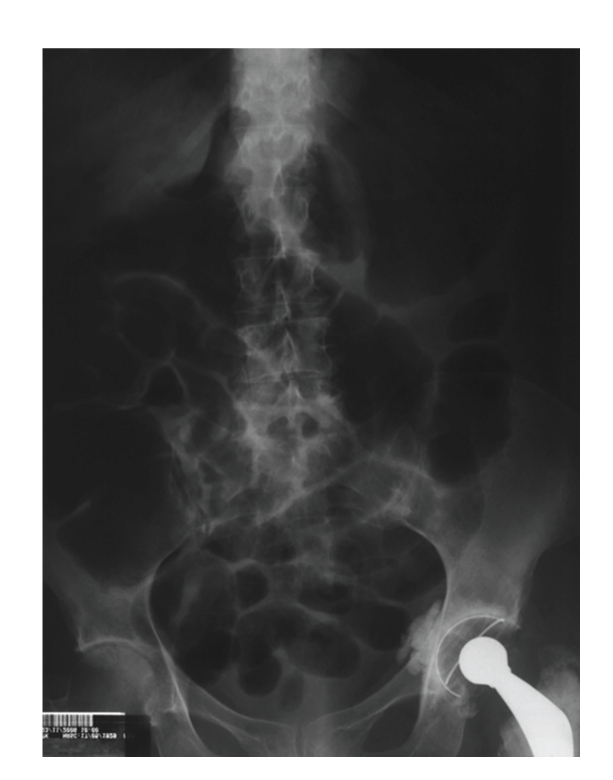
Abdomen radiograph showing the presence of high level of air fluid in the bowel loops.

On the basis of the aforementioned symptoms, the subject was submitted to hip arthroplasty for introduction of prosthesis in the left femur. The surgery lasted 4 hours. The anesthesiologist referred that the patient developed pronounced, hard-to-control hypotension (78 × 44 mmHg) within the first hour of the procedure, and remained hypotensive throughout the surgery. The arterial blood pressure of the patient in the preoperative period was 130 × 70 mmHg. Twelve hours postoperatively, the patient was still fasted and begun to complain of abdominal discomfort and nausea. Physical examination revealed good general health conditions, BP = 130 × 70 mmHg, and HR = 80 bpm, and the patient denied pain upon abdominal palpation. On the following day (24 hours postoperatively), the patient progressed with oliguria (530 mL/24 hours) and deteriorated general state, with onset of major abdominal pain accompanied by discomfort upon palpation. She was transferred to the Intensive Care Unit (ICU), where mental confusion, excess sudoresis, cyanotic extremities, pronounced hypotension (60 × 30 mmHg), and pale mucosa were noted, with evident signs of circulatory shock. Her APACHE II score was 31 [9]. The patient was intubated and placed under mechanical ventilation. In addition, vigorous fluid replacement with 2000 mL of 0.9% saline and 500 mL of starch was carried out. Examination upon ICU admission revealed severe metabolic acidosis (pH = 7.3, PaO_2_ = 65.9 mmHg, PaCO_2_ = 29.6 mmHg, and HCO_3_ = 7.8), with serum lactate of 19.8 mmol/L. Red blood cell, white blood cell, and platelet counts were 1.92 × 10^6^/mm^3^, 33.1 × 10^6^/mm^3^, and 154 × 10^6^/mm^3^, respectively; hemoglobin was 6.2 g/dL. Plasma biochemical tests furnished glycemia = 25 mg/dL, Na = 123 mmol/L, K = 5.3 mmol/L, and creatinine = 2.3 mg/dL. Due to circulatory instability, the patient was submitted to hemodynamic monitoring with Swan-Ganz catheter, which evidenced alteration in the main parameters (cardiac output = 3.9 mL/min/m^2^, pulmonary capillary pressure = 36 mmHg, and systemic vascular resistance = 800 din/s/cm^5^), thereby characterizing a septic shock with a clear left ventricular dysfunction component. Because the abdomen radiograph demonstrated a high level of air fluid in the bowel loops, NOMI was suspected (Figure 2). 

**Figure 3:**
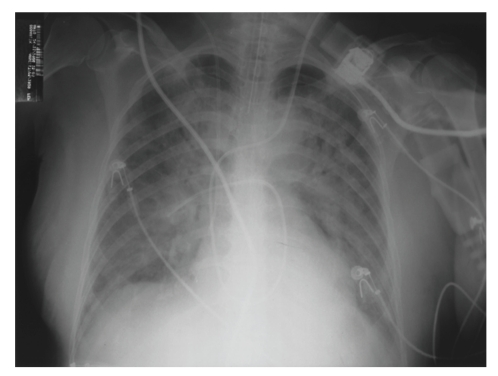
Chest radiograph showing bilateral pulmonary parenchymal infiltrate compatible with ARDS.

The patient was taken to the operation room, where exploratory laparotomy detected an 80 cm-length necrosis of the terminal ileum and cecum. A 100 cm bowel resection was performed. Upon return to the ICU, the individual persisted with hemodynamic instability, despite administration of increasing doses of noradrenaline. Acute respiratory distress syndrome (ARDS) developed (Figure 3), with disseminated intravascular coagulation. The patient died on the 4th postoperative day. Macroscopic analysis of the surgical specimen showed an apparent vascular patency and the absence of thrombi or atheromatous plaques within the vascular trunks or arteries and veins of smaller caliber. Moreover, there were no stenosis areas in the arterial network around the infarcted area.

## 3. Discussion

Ende was the first author to report on NOMI, in 1958 [10]. This condition is the consequence of a splanchnic arterial vasoconstriction resulting from a wide variety of clinical situations, which in turn cause reduced mesenteric blood supply. These events originate from decreased cardiac output due to ventricular dysfunction or arrhythmias, circulatory shock, digitalic intoxication, and use of vasosuppressors [5, 11]. Persistent vasoconstriction may lead to the formation of venous thrombosis, with consequent mesenteric necrosis and infarction. The mechanism through which bowel necrosis occurs during splanchnic hypoperfusion has not yet been fully elucidated. However, it seems that reperfusion of the affected location is a predisposing factor, which is partly mediated by oxygen free radicals and neutrophil adhesion to the endothelium of mesenteric venules. These neutrophils cause severe endothelial and microvascular lesion, thereby provoking Systemic Inflammatory Response Syndrome. It must be highlighted that NOMI is the result of a vasospasm (vasoconstriction) or hypoperfusion of the mesenteric vascular territory; whereas acute mesenteric ischemia is provoked by acute vascular obstruction (embolus). Several authors have detected NOMI as a complication of countless surgical procedures [12–14]. The present paper describes a state of transient hypotension during a surgical act on a patient with a history of arterial hypertension. 

Early diagnosis and prompt adequate treatment are key to the successful handling of NOMI patients. Experienced authors have indicated that diagnosis of this entity within 24 hours of its onset, associated with arteriography and vasodilating drugs, can significantly improve patient prognosis [15]. 

The angiographic diagnostic criteria for NOMI include narrowing of the origins of branches of the superior mesenteric artery, irregularities in the intestinal branches, spasm of the arcades, impaired filling of the intramural vessels, and slow flow with increased reflux of contrast into the abdominal aorta during selective injection of the superior mesenteric artery [16].

In this sense, prompt diagnosis of NOMI resides on the observation of three main points.

1. Recognition of patients with high risk of developing NOMI: individuals aged over 50 years with a history of congestive heart failure, recent myocardium infarction, hypovolemy, hypotension, or sepsis.
2. Recognition of occasional disparity between the presence of acute abdominal pain and absence of clinical signs upon abdominal examination. Delaying arteriography in these patients is one of the causes of significantly increased mortality.
3. Realization that waiting for a diagnosis based on definite clinical or radiograph abdominal symptoms, such as paralytic ileus and presence of air fluid in the intestinal loops, raises mortality. This is because these signs generally appear too late and are noted only after the onset of intestinal infarction or intestinal loop necrosis.

Abdominal pain may be absent in 15 to 25% of the NOMI patients, but distended belly and gastrointestinal bleeding, if present, may be premature. Many of the symptoms presented by the patients vary from nausea to vomiting, which might progress to rectal bleeding, hematemesis, intestinal obstruction, backache, large abdominal distension and, more seriously, circulatory shock. Serum leukocytes may exceed 15 × 10^3^/mm^3^ in over 75% of the patients. Metabolic acidosis occurs in 50% of the cases [6, 8]. Treatment must focus on the indication of early mesenteric arteriography or CT scan with computed tomography angiography (CTA) [17], removal or attenuation of the triggering factor (congestive heart failure, hypovolmy, sepsis), and promotion of mesenteric irrigation by means of hemodynamic stabilization (intravenous fluids and the careful use of vasoactive medication). Additonally, some authors advocate the use of vasodilators, such as papaverine [18], but the experience with its use in these patients remains limited. If intestinal loop necrosis is suspected, surgery should not be delayed. Prompt intensive care should be provided. Invasive hemodynamic monitoring with guided conduct is crucial to favorable patient prognosis. It is important to emphasize the severity of the present case. The patient was transferred to the intensive care unit for hemodynamic stabilization (IV fluids and vasoactive drugs) and immediately underwent surgery. It has been suggested the hypothesis of a stenosis in a branch artery since the patient was elderly and had a history of essential hypertension. Considering the fast shock onset and the history of sustained hypotension in this previously hypertensive patient, the decision to start the operation procedure immediately, without further examinations, was a consensus decision of the whole team (intensivists and surgeons). The surgical findings and pathological study of the anatomical specimens have further confirmed the initial diagnosis of NOMI.

## 4. Conclusion

Early NOMI diagnosis and high degree of clinical suspicion are the most crucial factors in the prevention of fatal complications. The orthopedist, surgeon, and intensivist should have knowledge of this condition so that prompt diagnosis and adequate treatment are accomplished, thereby preventing a fatal outcome. Through this paper, we would like to deliver a clear message to the orthopedist and, again, the anesthesiologists, intensivists, and surgeons, about the possible occurrence of NOMI, even in elective minor surgeries.
